# Determinants of Abl kinase stability and drug binding thermodynamics: Role of regulatory domains and phosphorylation

**DOI:** 10.1002/pro.70791

**Published:** 2026-09-16

**Authors:** Alan Juárez‐Barragán, J. Alberto Escobar‐Cázares, Miguel A. Medina‐Gómez, Ileana Tobías‐Juárez, Axel Luviano, Enrique García‐Hernández

**Affiliations:** ^1^ Universidad Nacional Autónoma de México, Instituto de Química Ciudad Universitaria Ciudad de México Mexico; ^2^ Instituto de Fisiología Celular, Departamento de Biología Celular y del Desarrollo Universidad Nacional Autónoma de México Ciudad de México Mexico

**Keywords:** Abelson tyrosine kinase, activation loop phosphorylation, interlobe cooperativity, kinase inhibitor thermodynamics, structural stability

## Abstract

Activity dysregulation of Abl kinase is the primary cause for chronic myeloid leukemia (CML). Despite extensive characterization, the thermodynamic basis of Abl stability and inhibitor recognition remains incompletely understood. To characterize the cooperative folding organization of Abl and the communication between its structural units, we comprehensively characterized three Abl constructs: the isolated catalytic domain (Abl_CD_), the three‐domain regulatory construct (Abl_SH3SH2CD_), and its phosphorylated form (^PO4^Abl_SH3SH2CD_), using high‐resolution calorimetric techniques. DSC revealed that the catalytic domain behaves as a single cooperative unfolding unit, with the SH3–SH2 module in Abl_SH3SH2CD_ exerting a modest stabilizing effect. The catalytic domain decoupled into two transitions in ^PO4^Abl_SH3SH2CD_, an observation rationalized by the disruption of the interlobe structural bridge formed by the A‐loop in the unphosphorylated forms. Imatinib and dasatinib had opposite effects on this decoupling, providing calorimetric evidence for the distinct intradomain reorganizations induced by each inhibitor type. ITC showed that both inhibitors bind with distinct thermodynamic signatures, with a modest effect of the regulatory domains on inhibitor recognition. Imatinib binding involved net proton uptake, consistent with Asp381 protonation required for the DFG‐out transition, while dasatinib showed no proton linkage. Phosphorylation‐induced reduction in imatinib affinity was driven by a large loss of favorable enthalpy reflecting the energetic cost of displacing the ordered A‐loop, while dasatinib affinity remained largely unaffected. These results provide a thermodynamic rationale for the superior potency of dasatinib against activated Abl in CML cells, revealing mechanistic features of kinase regulation not accessible from structural data alone.

## 1 INTRODUCTION

Tyrosine kinases are key enzymes in cellular signal transduction, regulating proliferation, differentiation, and survival (Buljan et al., 2020). Dysregulation of their activity due to mutations, chromosomal rearrangements, or aberrant expression is a recurrent feature of numerous diseases including cancer and immune disorders, making these proteins important therapeutic targets for the design of anticancer drugs (Cohen et al., 2021). One of the most extensively studied tyrosine kinases is human Abl (Colicelli, 2010; Hantschel & Superti‐Furga, 2004; Martins et al., 2024). The catalytic core of this emblematic enzyme consists of a CAP domain, an SH3 domain, an SH2 domain, and a bilobed catalytic domain. In the autoinhibited conformation of isoform 1b, Abl adopts a compact assembly in which the N‐terminal myristoyl moiety inserts into a hydrophobic pocket in the C‐terminal lobe of the kinase domain, inducing a kink in the αI helix that enables docking of the SH2 domain onto the C‐lobe, while the SH3 domain engages a PXXP motif within the SH2–catalytic domain linker, collectively stabilizing a closed conformation. Autoinhibition is further reinforced by positioning the conserved Asp‐Phe‐Gly (DFG) motif in the inactive DFG‐out conformation, with the activation loop (A‐loop) adopting a catalytically incompetent, conformationally flexible arrangement (Hantschel et al., 2003; Nagar et al., 2002, 2003; Shan et al., 2009).

Structural and biochemical studies have established that Abl dynamically switches between inactive DFG‐out conformation and active DFG‐in conformation. Four distinct conformational states of the Abl activation loop have been described: (i) an inactive state in which the A‐loop adopts a closed conformation with the DFG‐ in the out position and Tyr393 unphosphorylated (Nagar et al., 2002); (ii) an alternative inactive state, termed the Src‐like inactive conformation, characterized by a DFG‐in conformation but with the αC helix displaced outward, and with the Lys290‐Glu286 salt bridge required for catalysis disrupted (Levinson et al., 2006); (iii) the fully active state, in which phosphorylation of Tyr393 promotes the DFG‐in conformation and stabilizes the A‐loop in an extended conformation anchored to the C‐lobe through a network of electrostatic interactions (La Sala et al., 2016; Tokarski et al., 2006; Xie et al., 2020); and (iv) an alternative active state in which the A‐loop adopts an open DFG‐in conformation despite the absence of Tyr393 phosphorylation, reflecting the intrinsic conformational flexibility of the kinase and the partial decoupling between phosphorylation and structural activation (Tokarski et al., 2006).

The medical significance of Abl regulation is underscored by the chromosomal translocation t(9;22)(q34;q11), which generates the Philadelphia chromosome encoding the Bcr‐Abl fusion oncoprotein that drives chronic myeloid leukemia (CML) (Rowley, 1973). In Bcr–Abl, autoinhibition is abolished by the loss of the N‐terminal myristoylated CAP region and by constitutive oligomerization mediated by the Bcr coiled‐coil domain, maintaining the enzyme in a constitutively activated state (Hantschel & Superti‐Furga, 2004). Pharmacological targeting of Bcr–Abl has transformed CML therapy through multiple generations of inhibitors, with the above conformational states directly determining the mode of inhibitor recognition. Imatinib, the first FDA‐approved inhibitor, binds to the inactive DFG‐out conformation (type II inhibitor) (Nagar et al., 2002). Second‐generation inhibitors exhibit higher potency and expanded activity against imatinib‐resistant mutants, including the type I inhibitors dasatinib and bosutinib, which bind to the active DFG‐in conformation, and the type II inhibitor nilotinib (Weisberg et al., 2005). Ponatinib, a third‐generation type II inhibitor, was specifically designed to overcome the gatekeeper T315I mutation (Cortes et al., 2013). More recently, asciminib has emerged as a first‐in‐class allosteric inhibitor that binds to the myristoyl pocket, stabilizing the inactive conformation without engaging the ATP‐binding site (Schoepfer et al., 2018). However, despite this rich pharmacological and structural landscape, the thermodynamic basis of Abl stability and inhibitor recognition remains largely unexplored.

In particular, the energetic basis of the cooperative folding organization and molecular recognition ability of the enzyme, the communication between its regulatory elements, and how these properties are modulated by phosphorylation have not been systematically addressed. These questions are best approached using constructs that recapitulate the regulatory architecture of the oncogenic enzyme in vivo: the three‐domain SH3–SH2–catalytic domain construct (Abl_SH3SH2CD_) and its phosphorylated form (^PO4^Abl_SH3SH2CD_), which, lacking the N‐terminal myristoyl group, closely mimic the constitutively active core of Bcr‐Abl in CML cells. High‐resolution calorimetric techniques are uniquely suited to address these questions, as they provide direct access to the energetic underpinnings of structural stability and molecular recognition that are not accessible by structural or dynamic methods alone. The use of imatinib (IMA) and dasatinib (DAS) as molecular probes of the inactive and active conformations of the kinase, respectively, adds a further dimension that allows the calorimetric data to report on the conformational state of the catalytic domain in each construct and on the communication between regulatory elements. Comparison with the isolated catalytic domain (Abl_CD_) provides an additional axis to evaluate the specific contribution of the regulatory domains. Here we report a comprehensive calorimetric characterization of these three Abl constructs that revealed novel thermodynamic features of kinase cooperativity, phosphorylation‐induced interlobe decoupling, and inhibitor recognition, providing a quantitative energetic framework for understanding the differential potency of type I and type II inhibitors against the activated form of the enzyme.

## 2 RESULTS

We characterized the thermal unfolding behavior and ligand‐binding energetics of Abl_CD_, the three‐domain regulatory construct Abl_SH3SH2CD_, and its phosphorylated form ^PO4^Abl_SH3SH2CD_ using differential scanning (DSC) and isothermal titration (ITC) calorimetry. DSC experiments were performed at pH 7.4, and ITC binding measurements were carried out at pH 7.4 and 30°C. For Abl_SH3SH2CD_, ITC measurements were additionally performed at pH 8.0 to assess proton‐linkage effects associated with inhibitor binding.

### 2.1 Thermal unfolding analysis of non‐phosphorylated Abl_CD_ and Abl_SH3SH2CD_ constructs

**FIGURE 1:**
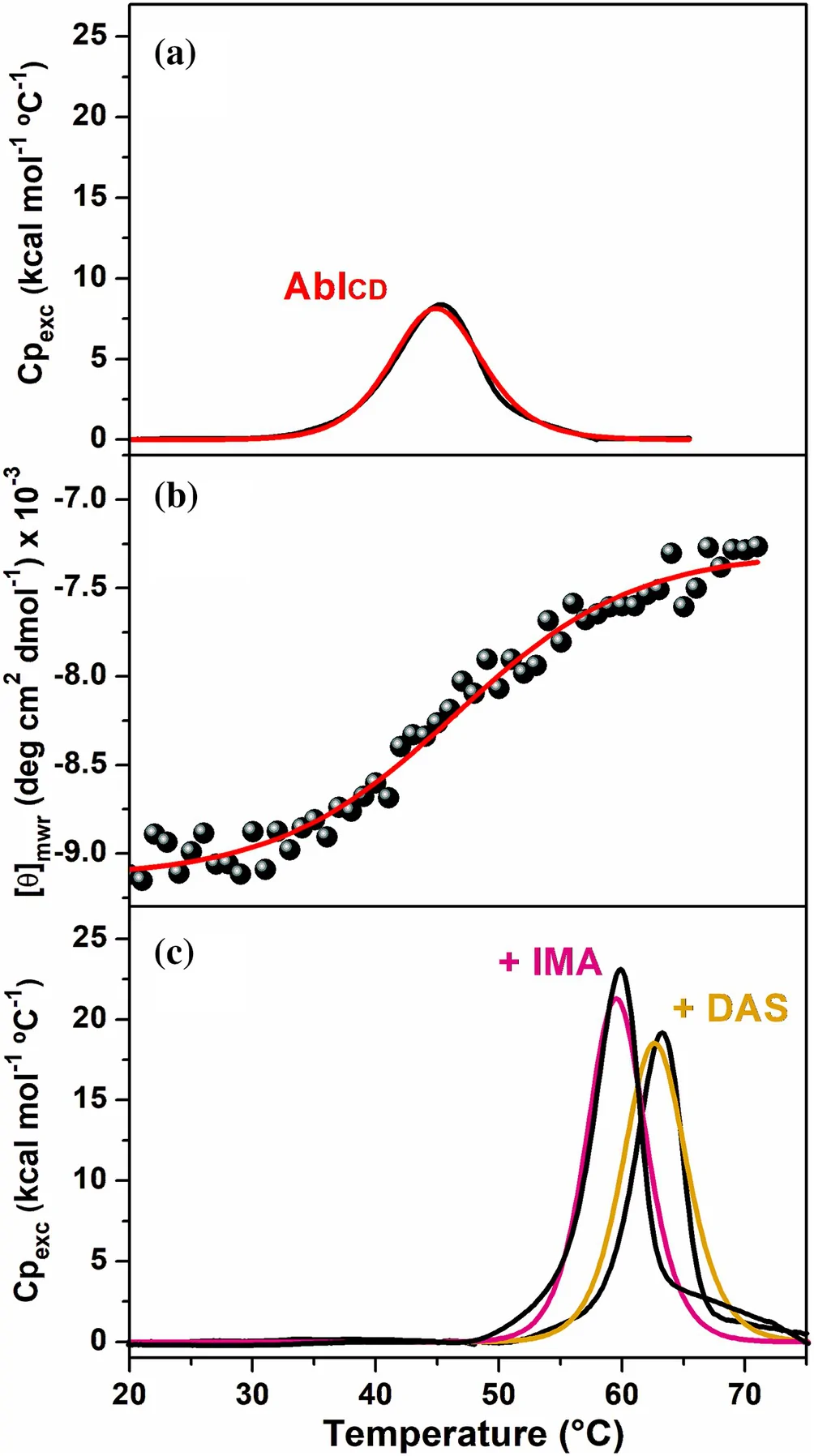
Thermal unfolding profiles of Abl_CD_. (a, b) DSC and CD profiles of the unfolding of the apo form of the domain, respectively. (c) DSC endotherms of the isolated domain in complex with IMA or DAS inhibitors. The DSC traces (black lines) for the catalytic domain (apo and inhibitor‐bound) were best fitted to a two‐state transition model (colored lines). The solid line shown in the CD profile represents a sigmoidal fit used to outline the secondary structure transition (*R* ^2^ = 0.980, *χ* ^2^/DoF = 8.94 × 10^3^ (deg cm^2^ dmol^−1^)^2^). All measurements were performed in 50 mM PIPES buffer, 150 mM NaCl, 5 mM MgCl_2_, 5% glycerol, pH 7.4.

The DSC thermogram of Abl_CD_ exhibited a single, symmetric peak with a Tm of ~45°C (Figure 1a). Far‐UV CD measurements revealed a gradual loss of secondary structure over a temperature range that closely overlapped with the calorimetric transition, confirming that the observed thermal effects corresponded to domain unfolding (Figure 1b). A second heating scan showed no recovery of the calorimetric trace, which was also observed by CD spectroscopy (data not shown), indicating fully irreversible behavior. Nevertheless, the unfolding endotherm of Abl_CD_ could be satisfactorily described using a two‐state pseudo‐equilibrium approximation. Similar behavior has been reported for other proteins with irreversible thermal transitions, where the process is assumed to proceed without significant kinetic deviation from a pseudo‐equilibrium condition (Sanchez‐Ruiz et al., 1988). To experimentally assess this condition, DSC measurements of Abl_CD_ were additionally performed at heating rates of 0.5 and 1.5°C/min (Figure S1). At the lowest scan rate, the transition shifted to lower temperature (*T*_*max*_ = 41.5°C), indicating a detectable kinetic contribution. In contrast, the thermograms obtained at 1.0 and 1.5°C/min were essentially superimposable, with *T*_*max*_ values of 45.2 and 45.3°C and calorimetric enthalpies of 79.9 and 79.0 kcal/mol, respectively. Thus, the scan rate used throughout this study (1.0°C/min) lay within a regime in which the apparent unfolding parameters are essentially insensitive to further increases in heating rate. Indeed, the shape of the Abl_CD_ endotherm at 1.0°C/min was consistent with a van't Hoff enthalpy (Δ*H*_*vH*_) of 84.2 kcal/mol, closely matching the experimental calorimetric enthalpy (Δ*H*_*vH*_/Δ*H*_*cal*_ = 1.05). Thus, although irreversibility precludes a rigorous thermodynamic analysis, the unfolding of the isolated domain can be reasonably described as a single‐transition process in which intermediate folding states are minimally populated. Figure 1c shows the unfolding endotherms of Abl_CD_ in the presence of 100 μM IMA or DAS. As observed, both inhibitors caused a significant increase in *Tm* and unfolding enthalpy (Table 1). Again, each transition could be adequately described by a two‐state model, confirming the cooperative nature of Abl_CD_ unfolding.

**TABLE 1:** Apparent calorimetric unfolding parameters of apo and drug‐complexed non‐phosphorylated Abl proteins^a^.

	*Tm*_1_ (°C)	Δ*H*_1_ (kcal/mol)	*Tm*_2_ (°C)	Δ*H*_2_ (kcal/mol)	*Tm*_3_ (°C)	Δ*H*_3_ (kcal/mol)
Abl_CD_						
apo^b^	45.0 ± 0.0	80.9 ± 0.2				
+ IMA^c^	59.6 ± 0.0	136.9 ± 0.7				
+ DAS^c^	62.7 ± 0.1	128.9 ± 1.0				
Abl_SH3SH2CD_						
apo^b^	49.6 ± 0.0	116.5 ± 0.6	64.1 ± 0.2	64.6 ± 0.8	78.3 ± 0.2	59.8 ± 0.9
+ IMA^c^	56.2 ± 0.0	140.6 ± 0.3	67.3 ± 0.1	65.2 ± 0.5	79.3 ± 0.2	54.6 ± 0.6
+ DAS^c^	57.6 ± 0.0	127.6 ± 0.7	68.3 ± 0.3	67.7 ± 1.2	80.1 ± 0.3	60.5 ± 1.3

^a^ All measurements were performed in a 50 mM PIPES buffer, 150 mM NaCl, 5 mM MgCl2, 5% glycerol, pH 7.4.

^b^ Reported values and uncertainties correspond to the mean ± SD obtained from two independent DSC measurements.

^c^ Reported uncertainties correspond to the standard errors of the nonlinear deconvolution procedure applied to the individual DSC thermograms.

**FIGURE 2:**
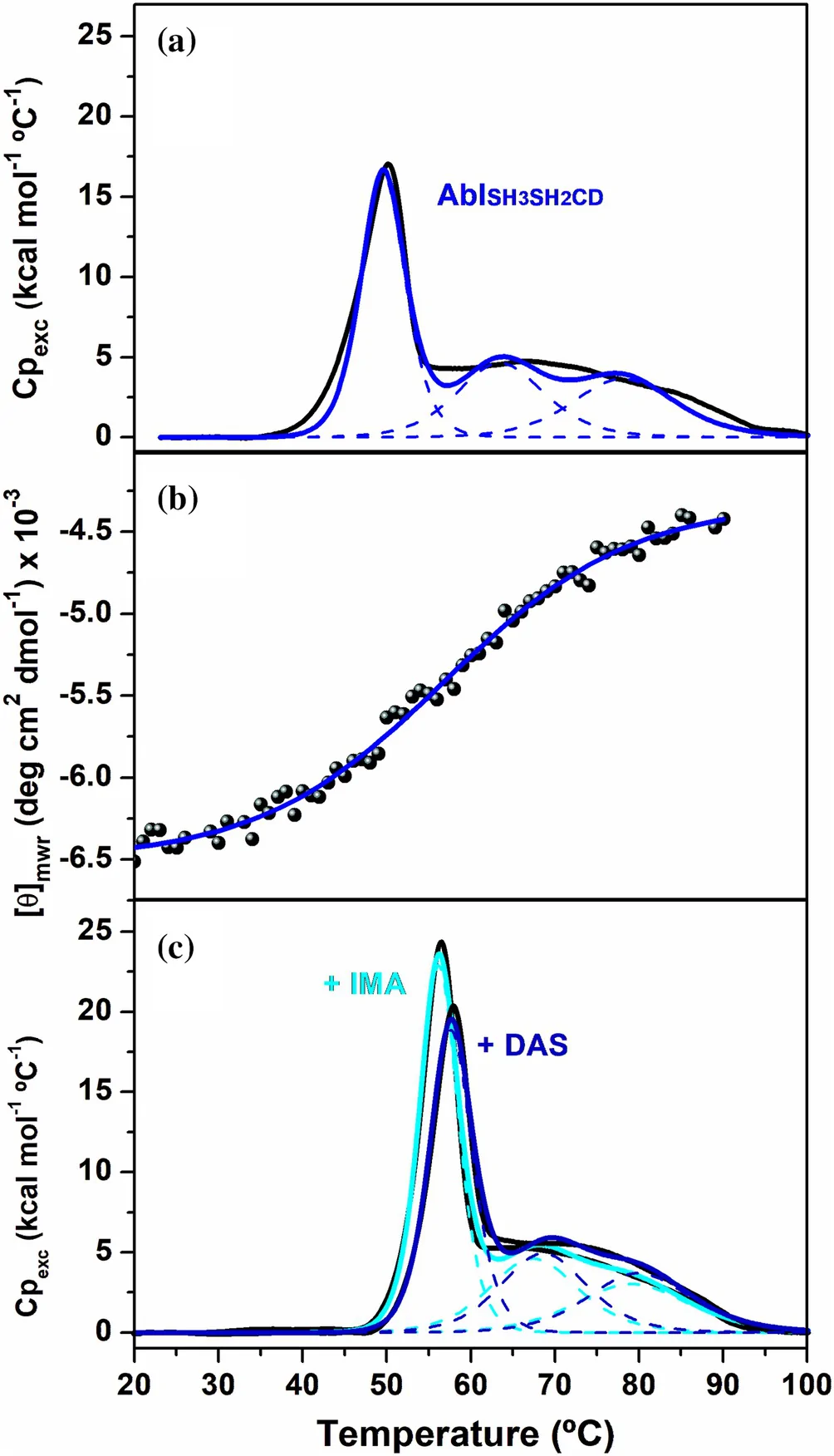
Thermal unfolding profiles of Abl_SH3SH2CD_. (a, b) DSC and CD profiles of the unfolding of the apo form of the three‐domain protein, respectively. (c) DSC endotherms of Abl_SH3SH2CD_ in complex with IMA or DAS inhibitors. The DSC traces (black lines) for the apo and inhibitor‐bound protein were best fitted to a model of three two‐state transitions (colored lines). The solid line shown in the CD profile represents a sigmoidal fit used to outline the secondary structure transition (*R* ^2^ = 0.993, *χ* ^2^/DoF = 3.43 × 10^3^ (deg cm^2^ dmol^−1^)^2^). All measurements were performed in 50 mM PIPES buffer, 150 mM NaCl, 5 mM MgCl_2_, 5% glycerol, pH 7.4.

The Abl_SH3SH2CD_ construct exhibited an unfolding endotherm with a greater number of apparent transitions (Figure 2a,b). A dominant low‐temperature peak was observed, followed by calorimetric effects distributed within a broad, asymmetric peak of relatively low intensity. Like the isolated catalytic domain, the three‐domain protein displayed fully irreversible thermal unfolding behavior. Nonetheless, the endotherm could be decomposed into three transitions centered at approximately 50, 64, and 78°C (Table 1). Both pharmacological inhibitors caused an increase in the temperature and enthalpy of the prominent low‐temperature peak, clearly indicating that this transition corresponds to the cooperative unfolding of the catalytic domain (Figure 2c, Table 1). This transition occurred at a *Tm* ~ 5°C higher than that of the isolated domain, suggesting a modest thermostabilizing effect of the regulatory domains on the catalytic domain. The broader, higher‐temperature peak was deconvoluted into two transitions, corresponding to the unfolding of the SH3 and SH2 regulatory domains (Corbi‐Verge et al., 2013). Supporting this interpretation, both transitions showed only minor changes in Tm and enthalpy upon binding of the orthosteric inhibitors.

### 2.2 Drug‐binding thermodynamic signatures of non‐phosphorylated Abl_CD_ and Abl_SH3SH2CD_ constructs

**FIGURE 3:**
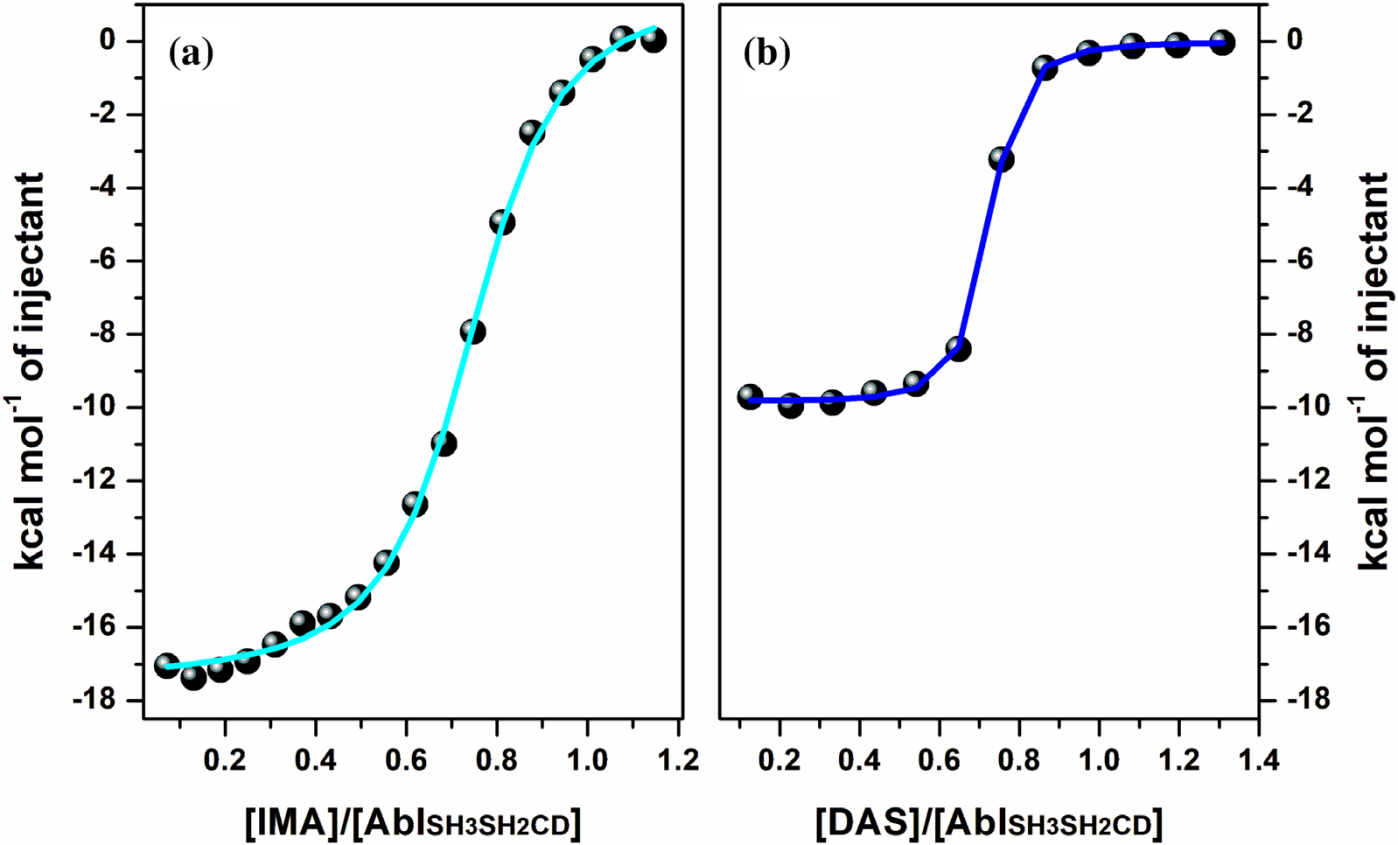
Binding isotherms of Abl_SH3SH2CD_. Representative ITC curves for the titration with (a) IMA and (b) DAS inhibitors. Experiments were conducted at 30°C in 50 mM PIPES containing 150 mM NaCl, 5 mM MgCl_2_, 5% glycerol.

Having established the cooperative unfolding properties of the two Abl constructs, we next investigated the interplay between regulatory domain assembly and inhibitor recognition by characterizing the binding of IMA and DAS, selective probes that trap the kinase in its inactive and active conformations, respectively. Figure 3 shows representative binding isotherms for the interaction of Abl_SH3SH2CD_ with the two orthosteric inhibitors at pH 7.4. For both drugs, binding was enthalpically driven, with DAS exhibiting an ~25‐fold higher affinity.

**FIGURE 4:**
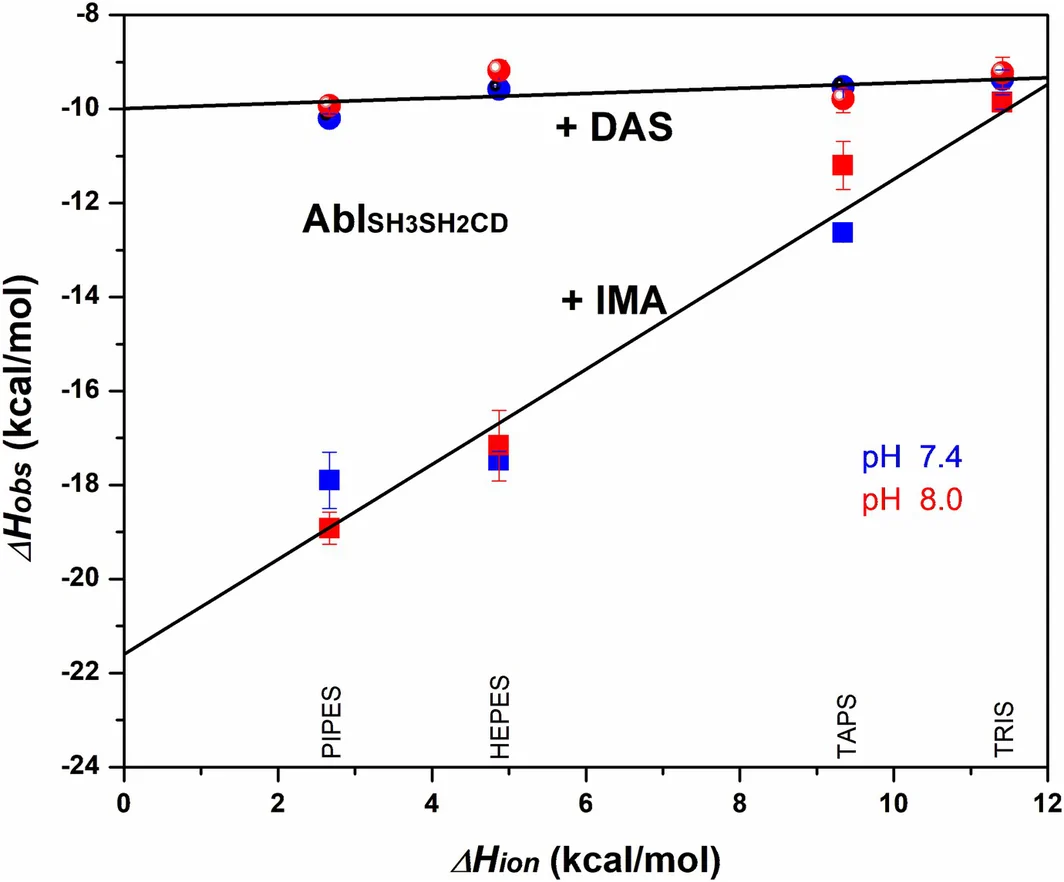
Proton‐linked energetics of IMA and DAS binding to Abl_SH3SH2CD_. Observed binding enthalpies (Δ*H*_*obs*_) at pH values of 7.4 (blue symbols) and pH 8.0 (red symbols) were measured in buffers with different proton ionization enthalpies (Δ*H*_*ion*_). Solid lines represent the best global linear fit to the measured enthalpies at both pH values for each inhibitor according to Δ*H*_*obs*_ = *η* Δ*H*_*ion*_ + Δ*H*_0_, where Δ*H*_0_ is the binding enthalpy free of buffer‐ionization effects (Bastos et al., 2023; Labra‐Núñez et al., 2021). IMA binding displayed a positive slope (*η* = 1.01 ± 0.07), consistent with the uptake of one proton during complex formation, whereas DAS showed a near‐zero slope (*η* = 0.05 ± 0.03), indicating no protonation linkage. Experiments were conducted at 30°C in 50 mM (PIPES, HEPES, TAPS or TRIS) buffer containing 150 mM NaCl, 5 mM MgCl_2_, 5% glycerol.

To evaluate protonation‐coupled effects during molecular recognition, binding measurements were performed in buffers with differing ionization enthalpies (Δ*H*_*ion*_). As shown in Figure 4, the observed binding enthalpy (Δ*H*_*obs*_) for IMA strongly depended on Δ*H*_*ion*_, following a trend quantitatively consistent with the uptake of one proton by a protein molecule upon inhibitor binding (*η* = 1.01 ± 0.07). In contrast, DAS binding was, within experimental error, buffer‐independent (*η* = 0.05 ± 0.03), indicating that no net protonation change occurred in the protein upon DAS binding. Virtually identical results for both inhibitors were obtained when binding was assessed at pH 8.0 (Figure 4). In a previous study on the isolated catalytic domain of Abl_CD_, it was shown that the conformational transition of the activation loop between the DFG‐in and DFG‐out states is coupled to proton exchange with the solvent through a change in the ionization state of Asp381, which exhibits an anomalously elevated *pKa* estimated at ~6.6 (Shan et al., 2009). In the inactive conformation, Asp381 becomes protonated to minimize electrostatic repulsion within the hydrophobic pocket in which it is buried. In the active conformation, it deprotonates and reorients toward the solvent, allowing coordination of the Mg^2+^ ion. Our findings are fully consistent with this mechanism, confirming that most of the free enzyme exists in the active conformation competent for DAS binding at physiological pH and therefore requires proton uptake to bind IMA.

Table 2 summarizes the thermodynamic signatures of IMA and DAS binding to Abl_SH3SH2CD_, corrected for buffer protonation effects. Once buffer ionization heats are taken into account, the IMA binding parameters obtained here agree well with previously reported values, although with a ~3‐fold weaker affinity (Kim et al., 2023). To our knowledge, no ITC data for DAS binding to Abl_SH3SH2CD_ have been previously reported. At both evaluated pH values, IMA binding enthalpy was approximately twice as exothermic as that of DAS. However, DAS binding was nearly isoentropic, whereas IMA binding involved a highly unfavorable entropy contribution. As a result, DAS binding was nearly 2 kcal/mol more exergonic than that of IMA. These contrasting thermodynamic signatures suggest that IMA binding is accompanied by a greater loss of conformational degrees of freedom than DAS binding, likely reflecting conformational changes associated with stabilizing the DFG‐out inactive state of the kinase domain. It is also worth noting that the affinity of DAS decreased ~3‐fold at pH 8.0 compared to pH 7.4, despite its negligible proton linkage, whereas no equivalent pH dependence was observed for IMA. Measurements at pH 7.4 therefore provide a more physiologically relevant reference for the binding potency of DAS in both normal and malignant hematopoietic cells.

**TABLE 2:** Intrinsic binding parameters of non‐phosphorylated Abl proteins to IMA and DAS inhibitors^a^.

System	pH	Δ*H*_b_ (kcal/mol)	Δ*G*_b_ (kcal/mol)	*T*Δ*S*_b_ (kcal/mol)	*K*_b_ (μM^−1^)	*K*_d_ (nM)
Abl_CD_						
+ IMA^b^	7.4	−23.3 ± 0.3	−10.0 ± 0.2	−13.4 ± 0.4	16.2 ± 6.3	61.7 ± 24.0
+ DAS	7.4	−9.9 ± 0.7	−10.9 ± 0.3	1.0 ± 0.7	74.8 ± 12.3	13.4 ± 2.4
Abl_SH3SH2CD_						
+ IMA^b^	7.4	−21.7 ± 0.4	−9.3 ± 0.3	−12.4 ± 1.0	5.1 ± 1.3	197 ± 56
+ DAS	7.4	−10.0 ± 0.2	−11.2 ± 0.4	1.2 ± 0.2	118.8 ± 27.0	8 ± 4
+ IMA^b^	8.0	−21.5 ± 0.5	−9.2 ± 0.1	−12.3 ± 0.6	4.3 ± 1.3	233 ± 70
+ DAS	8.0	−9.8 ± 0.4	−10.6 ± 0.4	0.8 ± 0.7	43.9 ± 17.0	23 ± 9

^a^ All measurements were performed at 30°C in 50 mM of the indicated buffer and pH, 150 mM NaCl, 5 mM MgCl_2_, 5% glycerol.

^b^ Binding enthalpies for IMA were corrected for buffer ionization effects to obtain the intrinsic binding values and the corresponding entropy contributions.

Table 2 also summarizes the thermodynamic signatures of drug recognition for Abl_CD_. Measurements were performed in PIPES buffer, which has the lowest ionization enthalpy of those used in this study, and the intrinsic binding enthalpy for IMA was obtained by correcting for the release of one proton from the buffer. Several studies have reported ITC parameters for the Abl_CD_‐IMA interaction under varying conditions, with *K*_*b*_ values ranging from 3.3 μM^−1^ (pH 6.4, 25°C) to 125 μM^−1^ (pH 7.1, 25°C). Our value falls within this range and is in close agreement with the value reported by Kim et al. (2023). In that study, as well as in Seeliger et al. (2007), the binding enthalpy is in good agreement with our buffer‐corrected value. In contrast, the ionization‐corrected enthalpy reported by Xie et al. (2020) is approximately half of these values despite a similar binding affinity, a discrepancy whose origin is unclear. For DAS, Liu et al. (2020) reported a ~7‐fold higher calorimetric association constant (*K*_*b*_ ≈ 500 μM^−1^) than our value at pH 7.4 and an ~11‐fold higher value than that measured at pH 8.0, although the measurements were performed at the same temperature (30°C) but under otherwise different experimental conditions, most notably at pH 6.4.

Comparison of the Abl_SH3SH2CD_ and Abl_CD_ data in Table 2 revealed that the isolated domain retains the overall thermodynamic features observed for the Abl_SH3SH2CD_ construct, with a strong enthalpic contribution for IMA binding and a nearly isoentropic signature for DAS. However, due to modest enthalpy–entropy compensatory changes, the relative binding advantage of DAS over IMA, as reflected in the magnitude of *K*_*b*_, decreases from ~23‐fold in Abl_SH3SH2CD_ to ~5‐fold in Abl_CD_.

### 2.3 Effect of activation loop phosphorylation on Abl_SH3SH2CD_ stability and drug binding

**FIGURE 5:**
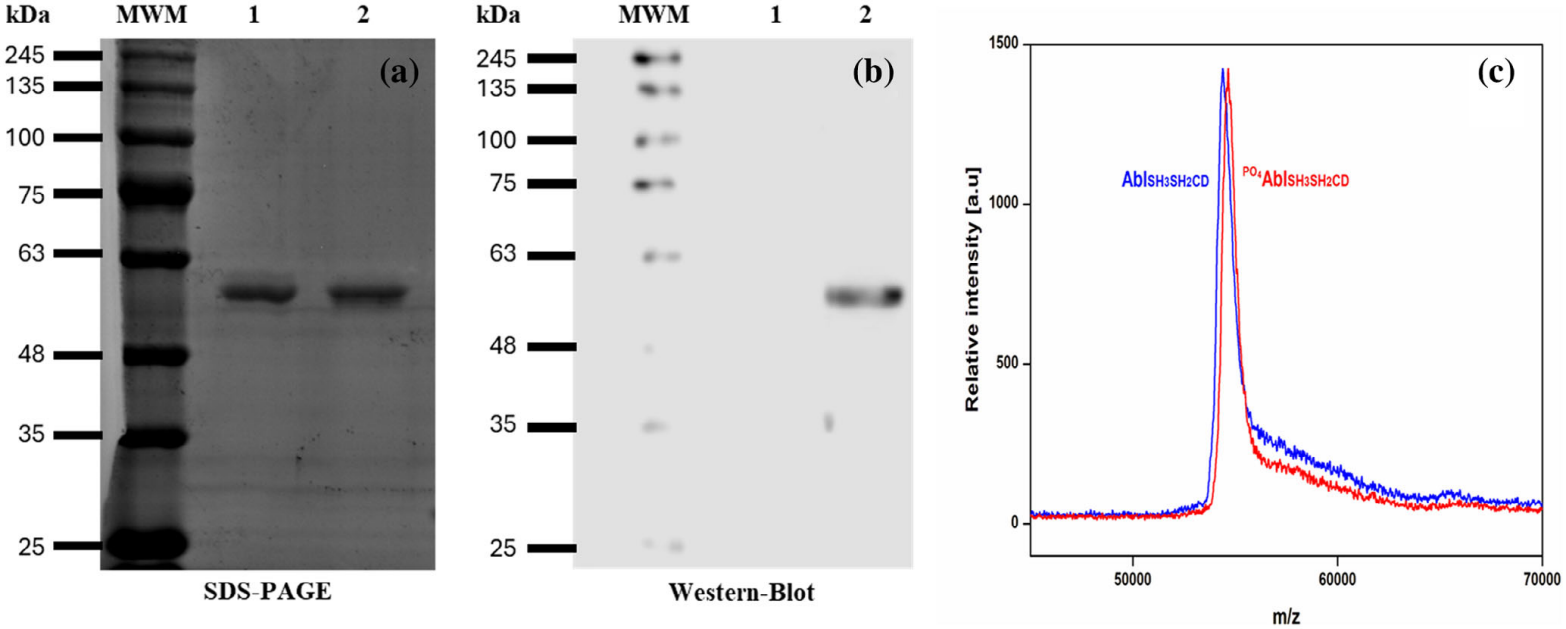
Phosphorylation of Abl_SH3SH2CD_. (a) SDS‐PAGE. (b) Western‐Blot analysis using an anti–phospho‐Tyr393 antibody. Lane 1: Abl_SH3SH2CD_. Lane 2: ^PO4^Abl_SH3SH2CD_. MWM: molecular weight marker. (c) MALDI‐TOF mass spectrometry. The determined molecular weights for Abl_SH3SH2CD_ and ^PO4^Abl_SH3SH2CD_ were 54,378 and 54,653 Da, respectively, corresponding to a mass difference of ~275 Da, consistent with the incorporation of three phosphate groups.

Phosphorylation of Tyr393 shifts Abl from a closed, autoinhibited state to an open and catalytically competent conformation. To examine how this activation step influences the unfolding and drug‐binding properties of Abl, we generated a phosphorylated form of Abl_SH3SH2CD_ (^PO4^Abl_SH3SH2CD_) by incubating the protein with MgATP for 1 h under autophosphorylation conditions. To confirm activation loop phosphorylation, samples were probed with an anti–phospho‐Tyr393 antibody, which revealed an immunoreactive band at the expected molecular weight, absent in the non‐phosphorylated control (Figure 5). In addition, intact‐protein MALDI‐TOF mass spectrometry showed an increase of ~275 Da for ^PO4^Abl_SH3SH2CD_ relative to Abl_SH3SH2CD_. This shift is compatible with the incorporation of three phosphate groups, given the expected mass increase of ~240 Da and the experimental uncertainty associated with mass determination of intact proteins of this size. The MALDI‐TOF spectra displayed a single well‐defined molecular ion peak, with no evidence of distinct mass populations that would suggest substantial phosphorylation heterogeneity. Taken together, these results confirmed the purity and structural integrity of the protein preparation, as well as phosphorylation of the activation‐loop residue Tyr393, a molecular hallmark of the active conformation of the kinase catalytic domain.

**FIGURE 6:**
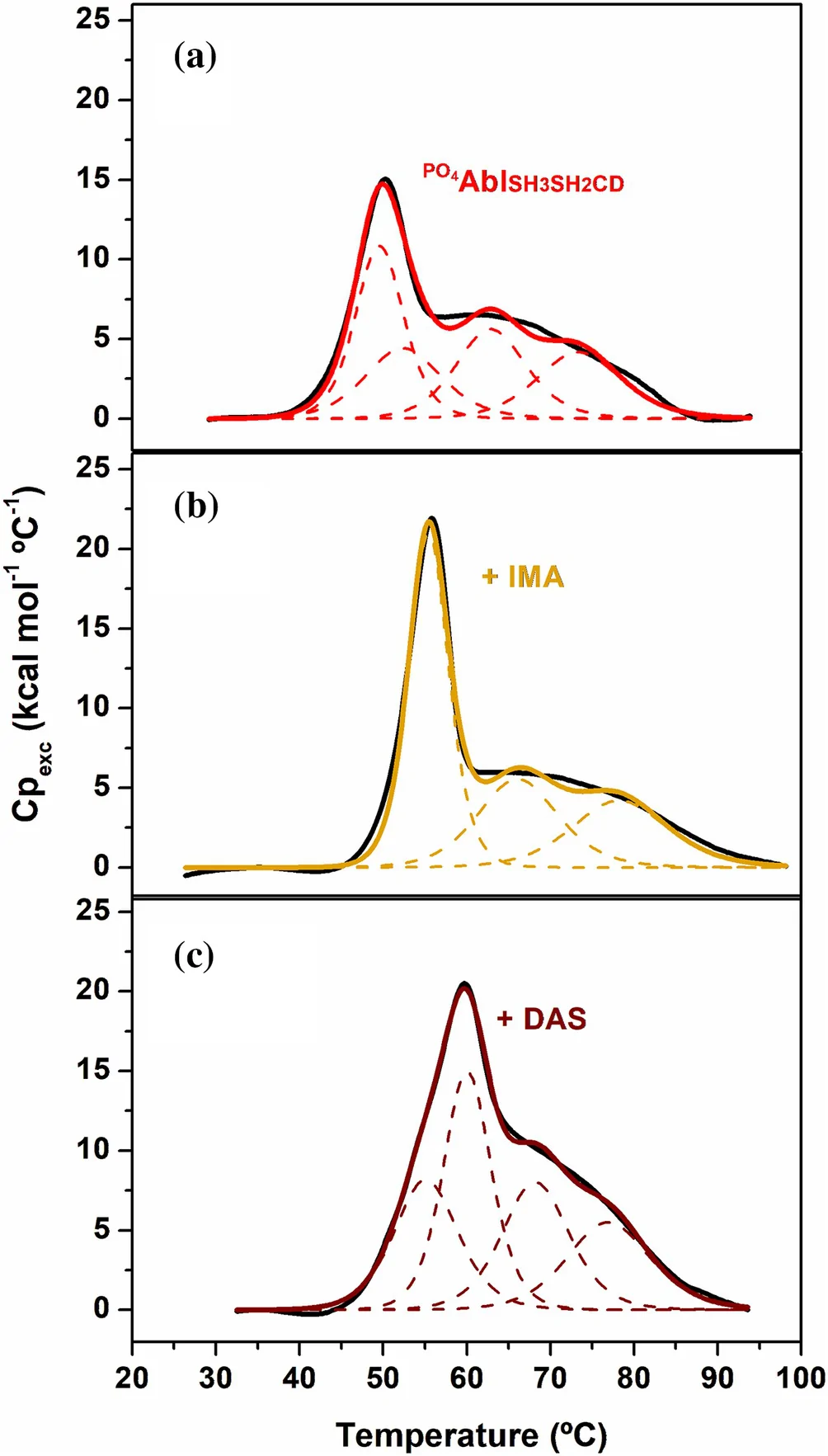
Thermal unfolding profiles of _PO4_Abl_SH3SH2CD_. DSC of the phosphorylated three‐domain protein (a) in the apo form and complexed with (b) IMA or (c) DAS. The DSC traces (black lines) for the apo and DAS‐bound protein were fitted to a model of four two‐state transitions (colored lines), whereas that of the IMA‐bound protein was best described with a model of three two‐state transitions. All measurements were performed in 50 mM PIPES buffer, 150 mM NaCl, 5 mM MgCl_2_, 5% glycerol, pH 7.4.

The unfolding endotherms of ^PO4^Abl_SH3SH2CD_ in its apo form and in complex with IMA or DAS are shown in Figure 6. Similar to Abl_SH3SH2CD_, the phosphorylated protein displayed a multistep unfolding. However, at variance with Abl_CD_ and Abl_SH3SH2CD_, the peak corresponding to unfolding of the catalytic domain split into two partially resolved transitions, rather than a single cooperative unit. In the apo protein, these transitions occurred at 48.3 and 51.5°C with unfolding enthalpies of 94.4 and 60.7 kcal/mol, respectively (Table 3). The differential unfolding behavior in the presence of inhibitors further highlighted this effect (Figure 6b,c). Notably, IMA binding restored the cooperative unfolding behavior observed for the non‐phosphorylated constructs (Figures 1 and 2). In contrast, two catalytic‐domain unfolding transitions remained evident in the presence of DAS, with *Tm* values of 55.2 and 60.1°C and enthalpy changes of 83.7 and 115.0 kcal/mol, respectively (Table 3). In this case, the transition with the largest enthalpy displayed the greatest increase in *Tm* upon inhibitor binding, suggesting preferential stabilization of the lobe harboring the active‐state binding determinants of DAS. As shown in Figure S2, the decomposition of the catalytic‐domain endotherm into two components in the apo and DAS‐bound form of ^PO4^Abl_SH3SH2CD_ was statistically supported by a markedly better fit quality compared to a three‐transition model. Taken together, this statistical criterion and the inhibitor‐dependent behavior of the two components argue against an artifactual origin of the deconvolution and support their assignment to structurally distinct cooperative units within the catalytic domain, consistent with a phosphorylation‐induced decoupling.

**TABLE 3:** ^PO4^Abl_SH3SH2CD_ unfolding and binding parameters to IMA and DAS inhibitors^a^.

DSC	*Tm*_1_ (°C)	Δ*H*_1_ (kcal/mol)	*Tm*_2_ (°C)	Δ*H*_2_ (kcal/mol)	*Tm*_3_ (°C)	Δ*H*_3_ (kcal/mol)
apo^b^	48.3 ± 0.1	94.4 ± 0.8	63.3 ± 0.1	71.1 ± 0.5	75.2 ± 0.1	63.4 ± 0.5
	51.5 ± 0.2	60.7 ± 0.9				
+ IMA^c^	55.5 ± 0.0	134.2 ± 0.4	66.2 ± 0.1	71.2 ± 0.6	78.4 ± 0.2	64.2 ± 0.6
+ DAS^c^	55.2 ± 0.0	83.7 ± 0.4	68.3 ± 0.1	86.2 ± 0.3	77.0 ± 0.1	73.2 ± 0.4
	60.1 ± 0.0	115.0 ± 0.3				

ITC	Δ*H*_b_ (kcal/mol)	Δ*G*_b_ (kcal/mol)	*T*Δ*S*_b_ (kcal/mol)	*K*_b_ (μM^−1^)	*K*_d_ (nM)
+ IMA^d^	−6.4 ± 0.1	−7.9 ± 0.1	1.5 ± 0.2	0.5 ± 0.1	2014 ± 400
+ DAS	−16.3 ± 0.6	−10.8 ± 0.1	−5.4 ± 0.6	66.1 ± 6.2	15 ± 1

^a^ All measurements were performed at 30°C in 50 mM PIPES buffer, 150 mM NaCl, 5 mM MgCl_2_, 5% glycerol, pH 7.4.

^b^ Reported values and uncertainties correspond to the mean ± SD obtained from at least two independent DSC measurements.

^c^ Reported uncertainties correspond to the standard errors of the nonlinear deconvolution procedure applied to the individual DSC thermograms.

^d^ The binding enthalpy for IMA was corrected for buffer ionization effects to obtain the intrinsic binding enthalpy and the corresponding entropy contribution.

In contrast to the catalytic domain behavior, the transitions corresponding to unfolding of the SH3–SH2 regulatory module, *Tm*_2_ and *Tm*_3_, remained mostly unaffected by phosphorylation or inhibitor binding (Table 3). This invariant behavior suggests that, once the catalytic domain has unfolded, the regulatory domains unfold independently and are not significantly stabilized by their interactions with the catalytic core.

A closer inspection of the apparent thermodynamic parameters in Tables 1 and 3 reveals some additional features worth noting. First, comparison of the apo forms across constructs shows that phosphorylation did not markedly modify the overall thermal stability of the catalytic domain. The *Tm* values of the two transitions in ^PO4^Abl_SH3SH2CD_ (48.3 and 51.5°C) narrowly straddled that of the single cooperative transition in Abl_SH3SH2CD_ (49.6°C). This observation indicates that the primary effect of phosphorylation was not a major change in thermal stability but rather a redistribution of cooperative interactions within the catalytic domain. This redistribution was accompanied by an increase in the total unfolding enthalpy of the catalytic domain in ^PO4^Abl_SH3SH2CD_ (94.4 + 60.7 = 155.1 kcal/mol) compared with that of the non‐phosphorylated construct (116.5 kcal/mol). Finally, the combined unfolding enthalpy of the SH3–SH2 module increased by ~25 kcal/mol from the apo ^PO4^Abl_SH3SH2CD_ to the DAS‐bound complex, an effect not observed in the IMA complex or in the non‐phosphorylated constructs. Overall, these selective enthalpic changes are consistent with the idea that DAS stabilized a distinct conformational ensemble of the phosphorylated enzyme.

**FIGURE 7:**
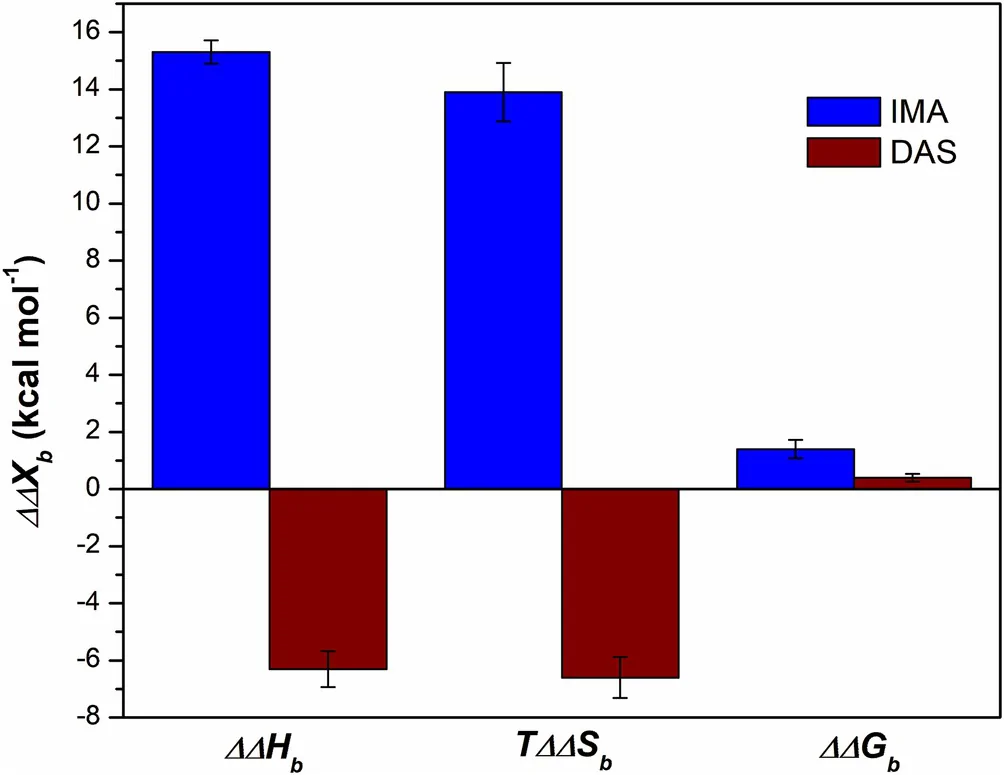
Differential thermodynamic effects of Tyr393 phosphorylation on the recognition of IMA and DAS by Abl_SH3SH2CD_. Differences in binding parameters were calculated as *∆∆X*_*b*_ = *∆X*_*b*_(^PO4^Abl_SH3SH2CD_) − *∆X*_*b*_(Abl_SH3SH2CD_), using the data reported in Tables 2 and 3. All measurements were performed in 50 mM PIPES buffer, 150 mM NaCl, 5 mM MgCl_2_, 5% glycerol, pH 7.4.

Finally, to quantify the effect of phosphorylation on recognition of the two orthosteric inhibitors, we determined the thermodynamic signatures of complex formation (Table 3). In agreement with previous reports (Seeliger et al., 2007), we found that the affinity for IMA was markedly reduced in the activated form (~10‐fold), whereas the affinity for DAS changed only modestly (~2‐fold reduction). However, as shown in Figure 7, the enthalpic and entropic contributions changed substantially for both complexes. In the case of IMA, binding to ^PO4^Abl_SH3SH2CD_ became considerably less exothermic compared to Abl_SH3SH2CD_, while the entropic contribution shifted from highly unfavorable to nearly isoentropic. In contrast, DAS binding to the phosphorylated enzyme became more exothermic and entropically less favorable.

The ITC results are consistent with the DSC data described above. The dramatic reduction in IMA binding enthalpy parallels the partial recovery of cooperative unfolding observed by DSC in the ^PO4^Abl_SH3SH2CD_–IMA complex, both suggesting that IMA can partially but not fully re‐establish the intradomain contacts characteristic of the inactive conformation. In contrast, the increased binding enthalpy of DAS in the phosphorylated construct is consistent with the selective enthalpic enhancement of the higher‐temperature catalytic transition observed by DSC, suggesting that DAS stabilizes a specific conformational state of the active catalytic domain. Taken together, the calorimetric data provide a quantitative framework linking Tyr393 phosphorylation, intradomain cooperativity, and inhibitor selectivity in Abl, with implications for understanding how the conformational state of the kinase shapes its pharmacological susceptibility.

## 3 DISCUSSION

The oncogenic Bcr‐Abl chimera protein was the first molecular target for which a drug was designed to treat cancer in a targeted manner. Despite the remarkable success of IMA, whose side effects are substantially milder than those of conventional chemotherapy, the emergence of resistance‐conferring mutations has sustained strong interest in developing new pharmacological strategies against this oncoprotein. At the same time, Abl represents a sophisticated regulatory switch controlling cell proliferation signaling pathways, whose conformational landscape and regulatory mechanisms remain under active investigation. In the present study, we have contributed an additional piece to the emerging picture of Abl function by thermodynamically characterizing underlying elements of its structural stability and drug recognition. To this end, we selected the conformation‐specific (type II) IMA and (type I) DAS inhibitors as molecular probes that drive the enzyme toward the inactive and active conformations, respectively. On the protein side, we simultaneously considered the isolated catalytic domain of Abl, which served as the basis for the earliest pharmacological designs, and compared it with the three‐domain construct incorporating the SH3–SH2 regulatory module, which is crucial for both autoinhibition and full activation of the enzyme (Grebien et al., 2011; La Sala et al., 2016; Lamontanara et al., 2014). Furthermore, to encompass an additional region of the complex conformational landscape of Abl associated with full kinase activation, we characterized the phosphorylated form of the three‐domain construct.

### 3.1 Phosphorylation‐induced interlobe decoupling of the Abl catalytic domain

The thermal unfolding data revealed that, despite its bilobal architecture, the catalytic domain behaves as a single cooperative folding unit under the conditions studied. Although thermal unfolding was irreversible, the scan‐rate analysis demonstrated that, at the experimental heating rate used in this study, the catalytic domain unfolded within a pseudo‐equilibrium regime. Together with the excellent agreement between the calorimetric and van't Hoff enthalpies and the satisfactory description of the endotherm by a two‐state model, these results support the cooperative nature of the unfolding transition with good confidence. However, phosphorylation, a milestone element for full activation of the enzyme, decoupled the catalytic domain into two distinct cooperative unfolding units, as evidenced by the partial resolution of the corresponding calorimetric peak into two transitions in ^PO4^Abl_SH3SH2CD_. Mechanistically consistent with this observation, DAS stabilized the decoupled state, whereas IMA reversed it, restoring a single cooperative unfolding unit.

The magnitude of the unfolding enthalpy of globular proteins has been related to the number of residues that become solvent‐exposed upon denaturation and is therefore considered an indicator of cooperative unfolding. In the available crystal structures (all of them solved in complex with one or more inhibitor molecules) of the Abl catalytic domain, approximately 270 residues are resolved. However, NMR measurements on the isolated catalytic domain reveal that ~25–30 of these residues, corresponding to the A‐loop and αC helix, are dynamically disordered in the apo state at physiological temperature (Xie et al., 2020). Using simple empirical relationships (Robertson & Murphy, 1997), the unfolding enthalpies of the catalytic domain in Abl_CD_ and Abl_SH3SH2CD_ are consistent with the unfolding of ~165 and ~210 residues, respectively, corresponding to roughly 65% and 82% of the structured residues. Interestingly, the two partially resolved transitions of the catalytic domain in ^PO4^Abl_SH3SH2CD_ together account for ~278 residues, consistent with the unfolding of the full complement of ordered residues in the domain. This is in line with the known ordering of the activation loop upon Tyr393 phosphorylation documented both crystallographically (Tokarski et al., 2006) and in solution by NMR (Xie et al., 2020), which would render it part of the cooperative unfolding unit. Taken together, these estimates indicate a progressive increase in the extent of ordered structure within the catalytic domain: from the isolated construct to the regulatory domain‐bound form and further upon phosphorylation.

A structural basis for the inhibitor‐dependent reversal of the phosphorylation‐induced decoupling is suggested by comparison of the crystal structures of the Abl catalytic domain bound to IMA and DAS. In the IMA‐bound structure (PDB: 1IEP; Nagar et al., 2002), the A‐loop adopts the DFG‐out inactive conformation and folds back onto the active site, establishing contacts with residues of both the N‐ and C‐lobes and thereby acting as a structural bridge between them. In the phosphorylated DAS‐bound structure, these interlobe contacts are absent (PDB: 2GQG; Tokarski et al., 2006), since the A‐loop is extended and ordered away from the active site. According to molecular dynamics simulations of apo forms of Abl, this fully open conformation of the A‐loop is stabilized by the formation of an interaction network with structural motifs forming also part of the C‐lobe (La Sala et al., 2016). This structural contrast provides a direct molecular rationale for the calorimetric observations: by capturing the DFG‐out conformation, IMA restores the interlobe contacts mediated by the A‐loop, recovering a single cooperative unfolding unit; DAS, by recognizing the active DFG‐in conformation, does not re‐establish these contacts, and the two‐transition behavior characteristic of the phosphorylated enzyme is preserved. In agreement with this structural interpretation, the number of residues estimated from the unfolding enthalpies of the two transitions in ^PO4^Abl_SH3SH2CD_ (~176 and ~104, respectively) is in excellent agreement with the number of residues in the C‐lobe (~180) and N‐lobe (~100) of the catalytic domain, raising the possibility that the two calorimetric transitions reflect the independent unfolding of each lobe. Although this assignment remains speculative in the absence of direct structural data on the phosphorylated apo form of Abl_SH3SH2CD_, it is consistent with the structural and computational evidence described above and provides a testable hypothesis for future studies. In particular, hydrogen‐deuterium exchange mass spectrometry of the phosphorylated apo protein could provide residue‐level information to directly assess whether these two calorimetric transitions indeed correspond to the independent unfolding of the N‐ and C‐lobes.

To our knowledge, the phosphorylation‐induced energetic decoupling of the Abl catalytic domain revealed by our DSC data has not been previously documented for this kinase or for any other tyrosine kinase by direct thermodynamic measurements. Conversely, the inverse phenomenon (i.e., phosphorylation‐induced intradomain coupling) has been demonstrated for the serine/threonine kinases PKA, based on structural and catalytic activity data (Steichen et al., 2012), ERK2, based on HDX‐MS data and molecular dynamics simulations (Pegram et al., 2023), and WNK1, based on molecular dynamics simulations (Jonniya et al., 2019). These comparisons highlight that the effect of activation loop phosphorylation on interlobe coupling is not universal among protein kinases but rather depends on the conformational context of each enzyme.

### 3.2 Thermodynamic determinants of inhibitor recognition

One of the central determinants of drug potency is the affinity for its molecular target (Copeland, 2016). In this regard, the thermodynamic characterization of binding not only provides a quantitative measure in terms of the equilibrium constant (or the Gibbs free energy change) but also reveals, through the determination of enthalpy and entropy changes, the underlying contributions of non‐covalent interactions, solvent reorganization, and changes in conformational degrees of freedom (Freire, 2008; García‐Hernández & Hernández‐Arana, 1999).

According to our ITC results, the SH3–SH2 regulatory module exerts a modest effect on the recognition of IMA and DAS by the catalytic domain. Both Abl_CD_ and Abl_SH3SH2CD_ recognize IMA with an affinity driven by a large enthalpy change and a concomitantly unfavorable entropic contribution. In contrast, the higher‐affinity recognition of DAS is characterized by a moderate enthalpy change and a marginally favorable entropy. The direct interpretation of this difference is that IMA induces rearrangements in the apo form of the catalytic domain that lead to the formation of a greater number of interactions, reflected in both a larger enthalpy gain and a concomitant loss of conformational degrees of freedom. This inference is consistent with the DSC data, in the sense that the positioning of the A‐loop over the catalytic site generates a structural bridge between the two lobes of the catalytic domain that contributes additional interaction surface.

The modest but systematic differences in binding affinity between Abl_CD_ and Abl_SH3SH2CD_ are mechanistically informative. The three‐domain construct used in this study lacks both the N‐terminal myristoyl group and the N‐cap region, and therefore adopts a disassembled configuration in which the SH2 domain interacts with the N‐lobe of the catalytic domain (PDB: 1OPL) (Grebien et al., 2011; Lamontanara et al., 2014). This interaction is known to stabilize the active, DFG‐in conformation of the catalytic domain. The opposing effects on IMA and DAS affinity observed upon addition of the SH3–SH2 module are consistent with this scenario: IMA, which captures the DFG‐out inactive conformation, undergoes an increased energetic penalty when the SH2 domain pre‐organizes the catalytic domain toward the active state, resulting in reduced apparent affinity; DAS, which recognizes the DFG‐in active conformation, benefits from this pre‐organization, resulting in increased apparent affinity. The net effect is a ~4.6‐fold shift in the relative binding advantage of DAS over IMA, as reflected in the *K*_*b*_ ratio, from ~5‐fold in Abl_CD_ to ~23‐fold in Abl_SH3SH2CD_.

The thermodynamic signatures of IMA and DAS binding also differ in their proton linkage, a property first characterized by Shan et al. through fluorescence‐based binding kinetics measurements (Shan et al., 2009). IMA binding is accompanied by the net uptake of one proton by the protein, whereas DAS binding shows no detectable proton exchange. This difference is mechanistically consistent with the distinct conformational changes induced by each inhibitor. Although Asp381 of the DFG motif has an anomalously high pKa, it remains predominantly deprotonated at pH 7.4, favoring the DFG‐in conformation in which Asp381 is solvent‐exposed and competent to coordinate the catalytic Mg^2+^. This picture is consistent with NMR data showing that the isolated catalytic domain exists predominantly in the active state at physiological temperature (Xie et al., 2020) and with our own ITC data. IMA binding therefore requires protonation of Asp381 to enable its burial into the hydrophobic pocket formed upon DFG flipping, in addition to the extensive reorganization associated with the DFG‐out transition. DAS, by recognizing the pre‐existing DFG‐in conformation, requires no protonation, and accordingly shows no proton linkage.

Despite the absence of detectable proton linkage, DAS binding exhibited a ~ 3‐fold decrease in affinity at pH 8.0 relative to pH 7.4. This observation indicates that the lack of net proton exchange upon binding does not necessarily imply complete pH independence of the binding free energy. Interestingly, Liu et al. (2020) reported a substantially higher affinity of DAS for the isolated Abl catalytic domain (*K*_*d*_ ≈ 2 nM), measured at the same temperature but under otherwise different experimental conditions, most notably at pH 6.4. Thus, the affinity of DAS progressively decreases from pH 6.4 to 7.4 and further to pH 8.0, although the larger differences between studies cannot be attributed solely to pH because of additional differences in experimental conditions. Nevertheless, the combined observations indicate that the molecular recognition of DAS is sensitive to the physicochemical environment despite the absence of detectable proton linkage. This behavior may reflect pH‐dependent changes in the electrostatic environment of the binding site and/or shifts in the complex conformational pre‐equilibria known to exist in Abl (Wilson et al., 2015), which can modulate ligand recognition without being directly coupled to proton uptake or release. Although the underlying mechanism remains to be established, these observations further support the use of pH 7.4 as the most physiologically relevant condition for characterizing inhibitor recognition by Abl.

The effect of phosphorylation on inhibitor binding thermodynamics is substantial and mechanistically asymmetric for IMA and DAS (Figure 7). The large enthalpy cost for IMA binding is consistent with the structural interpretation developed above: in the phosphorylated enzyme, the A‐loop is ordered in the extended, open conformation stabilized by interactions with the C‐lobe (La Sala et al., 2016; Tokarski et al., 2006; Xie et al., 2020). IMA must disrupt this interaction network to capture the DFG‐out conformation in which the A‐loop bridges the two lobes, a process that is enthalpically costly. The accompanying entropy gain likely reflects an increased conformational freedom of residues in the C‐lobe that accompanies the DFG‐out transition, consistent with local structural destabilization at the C‐lobe reported during IMA binding by unguided MD simulations and corroborated by HDX‐MS and NMR measurements (Ayaz et al., 2023).

For DAS, the thermodynamic picture is less straightforward. This inhibitor recognizes the active DFG‐in conformation, which is already thermodynamically favored at pH 7.4, and crystallographic comparison of the phosphorylated and unphosphorylated forms of the Abl catalytic domain bound to DAS reveals that the two structures are in general very similar (Nagar et al., 2002; Tokarski et al., 2006). The combination of a more favorable enthalpy change and an unfavorable entropy change is nevertheless consistent with the hypothesis that phosphorylation renders the enzyme‐DAS complex dynamically more restricted, allowing existing interactions to form with greater complementarity and persistence (enthalpy gain), while reducing the conformational degrees of freedom of the bound complex (entropic penalty). This interpretation, which would require validation by solution techniques sensitive to protein dynamics, is consistent with the DSC data documenting a more cooperative and structurally constrained catalytic domain in the phosphorylated enzyme. Taken together, the contrasting thermodynamic responses of IMA and DAS to phosphorylation provide a calorimetric fingerprint of the distinct conformational reorganizations induced by each inhibitor and underscore the thermodynamic basis for the differential sensitivity of type I and type II inhibitors to the activation state of the kinase.

The most directly comparable calorimetric study of Abl inhibitor binding is that of Kim et al. (2023), who characterized by ITC the interaction of IMA with Abl_CD_ and Abl_SH3SH2CD_, alongside the allosteric inhibitor asciminib and a myristoylated peptide (Kim et al., 2023). The affinities reported for IMA at pH 8.0 and 25°C are broadly consistent with ours, although an important methodological difference should be noted: Kim et al. performed their experiments in TRIS buffer without correcting for the heat of ionization of the buffer, which is substantial (ΔH_ion_ = 11.34 kcal/mol). Because IMA binding is accompanied by net proton uptake by the protein, the observed enthalpy in TRIS buffer includes a significant contribution from buffer ionization that shifts the apparent enthalpy toward less exothermic values. As a consequence, the binding enthalpy reported by Kim et al. is considerably less negative than ours, and the entropic contribution appears nearly neutral, a conclusion that would incorrectly suggest similar thermodynamic signatures for inhibitors of different binding modes. For the Src inhibitor 1, which binds the active conformation in a manner analogous to DAS, the binding constant was determined by FRET. This precludes a direct comparison of the full thermodynamic signature of active‐state inhibitors between the two studies (Toledo‐Stuardo et al., 2026). The present work therefore extends the thermodynamic characterization of Abl inhibitor binding in three important respects: it provides buffer‐corrected intrinsic thermodynamic parameters for both a type II inhibitor (IMA) and a type I inhibitor (DAS) across two protein constructs, it documents the dependence of binding thermodynamics on pH, and it characterizes for the first time to our knowledge the thermodynamic consequences of activation loop phosphorylation on inhibitor recognition.

## 4 CONCLUDING REMARKS

In summary, this study adds a thermodynamic dimension to the complex conformational landscape of Abl kinase and its recognition by pharmacological ligands. Using DSC, we showed that the catalytic domain behaves as a single cooperative unfolding unit in the isolated and regulatory domain‐bound forms of the enzyme, and that phosphorylation decouples this cooperative unit into two thermally independent transitions consistent with the independent unfolding of the N‐ and C‐lobes. The inhibitor‐dependent reversal of this decoupling provides calorimetric evidence for the role of the A‐loop as a structural bridge between the two lobes, and underscores the mechanistic distinction between type I and type II inhibitors at the level of intradomain cooperativity.

The ITC data revealed that the SH3–SH2 regulatory module, the protonation state of the DFG aspartate, and the phosphorylation state of the activation loop each contribute distinct thermodynamic signatures to inhibitor recognition. Most strikingly, the reduction in IMA affinity driven by a large loss of favorable binding enthalpy upon phosphorylation provides a thermodynamic rationale for the superior potency of DAS against activated BCR‐ABL. In CML, where BCR‐ABL is constitutively active and its activation loop predominantly phosphorylated, this condition substantially penalizes IMA binding while leaving DAS affinity largely intact. This thermodynamic asymmetry complements the structural explanation for the broader activity spectrum of DAS: the energetic cost of capturing the DFG‐out conformation in the phosphorylated enzyme is largely enthalpy‐driven and amounts to ~15 kcal/mol, only partially offset by a favorable entropy contribution. These results suggest that the phosphorylation state of the activation loop should be considered a relevant variable in the thermodynamic profiling of type II kinase inhibitors.

## 5 MATERIALS AND METHODS

### 5.1 Chemicals

Unless otherwise stated, all reagents were purchased from Sigma‐Aldrich. Buffers were prepared using Milli‐Q water and filtered through 0.22 μm membranes. Antibiotics for bacterial selection, including kanamycin and streptomycin, were purchased from GoldBio (Missouri, USA). All the equipment used for protein purification (columns and FPLC) was acquired from Cytiva (Massachusetts, USA). All reagents used for SDS–PAGE, immunoblotting, and chemiluminescent detection were of molecular biology grade. Imatinib was prepared as a concentrated stock solution in Milli‐Q water, whereas DAS was solubilized in Milli‐Q water with the aid of methanesulfonic acid to ensure complete dissolution.

### 5.2 Protein expression and purification

Numbering for the spliceform 1a of the human *ABL1* gene is used throughout the manuscript. The Abl_CD_ (residues 229–511 with a C‐terminal His_6_ tag) construct in pET28a‐TK‐ABL and the YopH phosphatase in pCDFDuet‐1‐YopH were kindly provided by Dr. John Kuriyan (University of California, Berkeley, USA). The Abl_SH3SH2CD_ sequence (residues 64–511) was commercially synthesized (Laragen, California, USA) and subcloned into pET28a between the NdeI and HindIII sites, introducing an N‐terminal His_6_ tag. *E. coli* BL21(DE3) cells were cotransformed with either ABL construct (Abl_CD_ or Abl_SH3SH2CD_) together with the YopH plasmid, ensuring a nonphosphorylated state during bacterial expression. Cultures were grown in LB medium supplemented with kanamycin (50 μg/mL) and streptomycin (50 μg/mL) (GoldBio, Missouri, USA) at 37°C until reaching an OD_600_ of 0.6–0.8. Protein expression was induced with 0.1 mM IPTG (GoldBio, Missouri, USA) for 18 h at 23°C. Cells were harvested by centrifugation (5000*g*, 10 min) and resuspended in lysis buffer (50 mM Tris–HCl pH 8.0, 150 mM NaCl, 5 mM MgCl_2_, 5% glycerol, 20 mM imidazole, 1 mM PMSF). After sonication on ice, the lysate was cleared by centrifugation (12,000*g*, 20 min), filtered (0.45 μm), and loaded onto a HisTrap HP Ni^2+^ affinity column (Cytiva, Massachusetts, USA). Bound protein was eluted with the same buffer containing 500 mM imidazole. The eluate was subsequently subjected to anion‐exchange chromatography on a HiTrap Q FF (Cytiva, Massachusetts, USA) and eluted with buffer containing 50 mM Tris–HCl pH 8.0, 1 M NaCl, 5 mM MgCl_2_, and 5% glycerol. The protein was then concentrated to 2 mL in SEC buffer (50 mM Tris–HCl pH 8.0, 150 mM NaCl) and applied to a HiLoad Superdex 75 pg. size‐exclusion column (Cytiva, Massachusetts, USA) for final purification. All chromatographic steps were performed using an ÄKTA Go system (Cytiva, Massachusetts, USA). Protein purity at each step was assessed by 12% SDS–PAGE using Laemmli buffer (50 mM Tris–HCl pH 6.8, 3% SDS, 1% β‐mercaptoethanol, 20% glycerol, 0.7% bromophenol blue), stained with Coomassie Brilliant Blue R‐250.

### 5.3 Phosphorylation and immunoblotting

Purified Abl_SH3SH2CD_ was subsequently phosphorylated in vitro to generate the activated form of the enzyme (^PO4^Abl_SH3SH2CD_) following a protocol similar to that reported previously (Lamontanara et al., 2014). Auto‐phosphorylation was carried out by incubating the protein at 25°C with 10 mM ATP and 5 mM MgCl_2_. The reaction was allowed to proceed for 1 h, a condition selected to achieve efficient phosphorylation of the activation‐loop residue Tyr393 while minimizing the progressive multisite autophosphorylation reported for prolonged incubations (Lamontanara et al., 2014). Following incubation, excess ATP was removed by buffer exchange. For immunoblotting, proteins solved by 12% SDS–PAGE gels were transferred onto nitrocellulose membrane and blocked with 5% non‐fat milk in TBS (50 mM Tris–HCl pH 7.4, 150 mM NaCl). Membranes were incubated with a monoclonal anti–phospho‐c‐Abl (Tyr393) antibody (Santa Cruz Biotechnology, Texas, USA, sc‐293,130) in a dilution of 1:1000, followed by HRP‐conjugated anti‐mouse IgG (1:5000). Signal detection was performed using the SuperSignal® West Femto substrate (Thermo Fisher Scientific Inc., Massachusetts, USA) and visualized with a C‐DiGit Blot Scanner (LICORbio, Nebraska, USA).

### 5.4 MALDI–TOF mass spectrometry

Protein identity and integrity were confirmed by MALDI–TOF mass spectrometry using a Microflex LTTM instrument (Bruker Daltonics, Massachusetts, USA) as described elsewhere (Leyva et al., 2019). Samples were diluted 1:10 in sterile deionized water and mixed with a saturated solution of Super‐DHB prepared in 0.1% TFA and 30% acetonitrile. Spectra were acquired in positive‐ion linear mode and analyzed with FlexAnalysis 3.0 software (Bruker Daltonics, Massachusetts, USA).

### 5.5 Circular dichroism (CD) spectroscopy

Far‐UV CD spectra were recorded on a JASCO J‐1500 spectropolarimeter (JASCO Inc., Maryland, USA) as described elsewhere (Arreguín‐Espinosa et al., 2001). Spectra were acquired from protein samples at ~0.2 mg/mL in 1 mM PIPES buffer, pH 7.4, containing 15 mM NaF, 0.5 mM MgCl_2_, and 0.5% glycerol, using a 0.1 cm path‐length cell. Each final spectrum represents the average of three accumulations. CD signals were normalized and reported as mean residue ellipticity ([*θ*]_*mrw*_): ${\left[\theta \right]}_{\mathrm{mrw}}=\frac{\mathrm{mrw}\times \theta \times 100}{C\times l}$, where *mrw* is the molecular weight of a mean residue, *θ* is the experimental ellipticity, *C* is the protein concentration in mg/mL and *l* is the path length in cm. Thermal unfolding was monitored from 20 to 90°C at a heating rate of 1°C/min.

### 5.6 Differential scanning calorimetry (DSC)

Thermal stability was assessed using a MicroCal VP‐capillary DSC system (Malvern Panalytical Ltd., Malvern, UK), as described previously (Medrano‐Cerano et al., 2024). Protein samples (~30 μM for Abl_CD_ and ~18 μM for Abl_SH3SH2CD_/^PO4^Abl_SH3SH2CD_), with or without 100 μM DAS or IMA, were heated from 15 to 110°C at a scan rate of 1°C/min. Buffer–buffer baselines were acquired under identical conditions before recording the signal from the protein solution and subtracted from the corresponding sample traces. All solutions were degassed for 10 min before each run. Resulting endotherms were fitted to independent transition‐unfolding models programmed in the MicroCal Origin v7.0 software (OriginLab, Massachusetts, USA). DSC experiments for the apo proteins were performed at least in duplicate. Reported values correspond to the mean ± SD from at least two independent measurements. For the complexes, reported uncertainties correspond to the standard errors obtained from nonlinear deconvolution of the individual DSC thermograms. All measurements were performed in a 50 mM PIPES buffer, 150 mM NaCl, 5 mM MgCl_2_, 5% glycerol, pH 7.4.

The van't Hoff enthalpy (*∆H*_*vH*_) was calculated from the unfolding endotherm as follows:

$$∆H_{\mathrm{vH}}=\frac{4RT_{\max}^{2}C_{p,\max}}{∆H_{\mathrm{cal}}},$$

where *T*_*max*_ is the temperature at the maximum of the endotherm, *C*_*p,max*_ is the maximum excess heat capacity at *T*_*max*_, and *∆H*_*cal*_ is the area under the baseline‐corrected endotherm.

### 5.7 Isothermal titration calorimetry (ITC)

Ligand‐protein binding thermodynamics was measured using a MicroCal™ iTC200 calorimeter (Malvern Panalytical Ltd., Malvern, UK) at 30°C as described previously (Salcedo et al., 2014). Protein concentrations ranged from 15 to 30 μM, and ligand concentrations were 150 μM for IMA and 100 μM for DAS. Titrations consisted of ~20 consecutive injections of ligand separated by 270 s, using a stirring rate of 750 rpm. Ligand dilution heats were determined under identical conditions and subtracted from raw data. All samples were degassed for 10 min before each experiment. Binding parameters were determined by using an identical and independent binding site model (Bastos et al., 2023; Leyva et al., 2019):

$$Q_{i}=\begin{array}{l} \frac{nM_{t}∆H_{b}V_{O}}{2}[1+\frac{L_{t}}{nM_{t}}+\frac{1}{nK_{b}M_{t}}\\ -\;\sqrt{{\left(1+\frac{L_{t}}{nM_{t}}+\frac{1}{nK_{b}M_{t}}\right)}^{2}-\frac{4L_{t}}{nM_{t}}}], \end{array}$$

where *Q*_*i*_ is the total normalized heat evolved per mol of ligand at the end of the *i* ^th^ injection, *K*_*b*_ is the binding constant, Δ*H*_*b*_ is the enthalpy change, *n* is the stoichiometry, *V*_*o*_ is the working volume of the cell, and *L*_*t*_ and *M*_*t*_ are the total ligand and macromolecule concentrations, respectively. The heat released in the *i* ^th^ injection is:

$$∆Q_{i}=Q_{i}+\frac{v_{i}\left(Q_{i}+Q_{i-1}\right)}{2V_{O}}-Q_{i-1}+q_{\mathrm{dil}},$$

where *v*_*i*_ is the aliquot volume added at injection *i* and *q*_*dil*_ is a fitting term introduced to account for experimentally uncorrected dilution heat effects. The non‐linear regression was carried out using the MicroCal Origin v7 software (OriginLab, Massachusetts, USA).

Proton exchange coupled to ligand binding was assessed by conducting calorimetric measurements with buffers of different ionization enthalpies (Δ*H*_*ion*_). If proton exchange occurs during complex formation, the observed enthalpy (Δ*H*_*obs*_) varies linearly with Δ*H*_*ion*_ according to the equation:

$$\Delta H_{\mathrm{obs}}=\eta \;\Delta H_{\mathrm{ion}}+\Delta H_{0},$$

where *η* is the number of protons exchanged, and Δ*H*_0_ is the intrinsic binding enthalpy corrected by buffer ionization effects (Labra‐Núñez et al., 2021). Experiments were performed in 50 mM buffers with *pKa* values near 7.5 and different (deprotonation) Δ*H*_*ion*_: TRIS (11.34 kcal/mol), TAPS (9.34 kcal/mol), HEPES (4.87 kcal/mol), or PIPES (2.67 kcal/mol), supplemented with 150 mM NaCl, 5 mM MgCl_2_, and 5% glycerol. All experiments were performed in triplicate, and reported values represent the mean ± standard deviation.

## AUTHOR CONTRIBUTIONS

**Axel Luviano:** Conceptualization; writing – review and editing; methodology; investigation; writing – original draft; formal analysis. **Ileana Tobías‐Juárez:** Writing – review and editing; methodology; investigation. **J. Alberto Escobar‐Cázares:** Investigation; methodology; writing – review and editing. **Miguel A. Medina‐Gómez:** Investigation; methodology; writing – review and editing. **Enrique García‐Hernández:** Conceptualization; writing – original draft; writing – review and editing; supervision; funding acquisition; formal analysis. **Alan Juárez‐Barragán:** Writing – review and editing; methodology; investigation; formal analysis; writing – original draft.

## CONFLICT OF INTEREST STATEMENT

The authors declare no conflicts of interest.

## Supporting information


**Figure S1.** DSC endotherms of Abl_CD_ as a function of scan rate. Endotherms were recorded at 0.5°C/min (olive green), 1.0°C/min (blue), and 1.5°C/min (red). The table summarizes *T*_*max*_ (maximum of each endotherm), Δ*H*_*cal*_ (area under the baseline‐corrected endotherm) and Δ*H*_*vH*_ (calculated from the shape of the endotherm assuming a two‐state transition model).
**Figure S2.** Comparison of three‐ and four‐transition deconvolution models for the DSC endotherms of ^PO4^Abl_SH3SH2CD_. (A, B) Apo form fitted to three‐ (*χ* ^2^/DoF = 9.0 × 10^5^) and four‐transition models (*χ* ^2^/DoF = 1.3 × 10^5^), respectively. (C, D) DAS‐bound form fitted to three‐ (*χ* ^2^/DoF = 2.3 × 10^6^) and four‐transition models (*χ* ^2^/DoF = 6.3 × 10^4^), respectively. In both cases, the four‐transition model provides a markedly superior description of the experimental data, supporting the assignment of the two low‐temperature transitions to structurally distinct cooperative units within the catalytic domain and the two high‐temperature transitions to the regulatory SH3 and SH2 domains.

## Data Availability

The data that support the findings of this study are available on request from the corresponding author.
