# Supplementary material for: Determinants of Abl kinase stability and drug binding thermodynamics: Role of regulatory domains and phosphorylation
Source: Protein Sci. 2026 Sep 16;35(10):e70791. doi: 10.1002/pro.70791 (PMC13580156; doi:10.1002/pro.70791)
Supplement: Supplementary file 1 [file PRO-35-e70791-s001.docx]

**Determinants of Abl Kinase Stability and Drug Binding Thermodynamics: Role of Regulatory Domains and Phosphorylation**

Alan Juárez-Barragán^1^, J. Alberto Escobar-Cázares^1^, Miguel A. Medina-Gómez^1^,

Ileana Tobías-Juárez^2^, Axel Luviano^1,^*, Enrique García-Hernández^1,^*

^1^ Universidad Nacional Autónoma de México, Instituto de Química, Ciudad Universitaria, Ciudad de México, 04510.

^2^ Instituto de Fisiología Celular, Departamento de Biología Celular y del Desarrollo, Universidad Nacional Autónoma de México, CDMX 04510, México.

**Figure S1. DSC endotherms of Abl_CD_ as a function of scan rate.** Endotherms were recorded at 0.5 °C/min (olive green), 1.0 °C/min (blue), and 1.5 °C/min (red). The Table summarizes *T_max_* (maximum of each endotherm), *ΔH_cal_* (area under the baseline-corrected endotherm) and *ΔH_vH_* (calculated from the shape of the endotherm assuming a two-state transition model).

**Figure S2. Comparison of three- and four-transition deconvolution models for the DSC endotherms of ^PO4^Abl_SH3SH2CD_. (A, B)** Apo form fitted to three- (χ²/DoF = 9.0 × 10⁵) and four-transition models (χ²/DoF = 1.3 × 10⁵), respectively. **(C, D)** DAS-bound form fitted to three- (χ²/DoF = 2.3 × 10⁶) and four-transition models (χ²/DoF = 6.3 × 10⁴), respectively. In both cases, the four-transition model provides a markedly superior description of the experimental data, supporting the assignment of the two low-temperature transitions to structurally distinct cooperative units within the catalytic domain and the two high-temperature transitions to the regulatory SH3 and SH2 domains.
